# Flt3 ligand treatment reduces enterovirus A71 lethality in mice with enhanced B cell responses

**DOI:** 10.1038/s41598-018-30631-2

**Published:** 2018-08-15

**Authors:** Yu-Wen Lin, Li-Chiu Wang, Chien-Kuo Lee, Shun-Hua Chen

**Affiliations:** 1 Institute of Biomedical Sciences, College of Medicine, National Cheng Kung University, Tainan, Taiwan; 2 Institute of Immunology, College of Medicine, National Taiwan University, Taipei, Taiwan; 3 Department of Microbiology and Immunology, College of Medicine, National Cheng Kung University, Tainan, Taiwan

## Abstract

Enterovirus A71 (EV-A71) infection can induce encephalitis, which causes death or long-term neurological sequelae, especially in young children. Using a murine infection model, we searched for anti-EV-A71 agents, because effective therapies are not available to control fatal infection. In EV-A71-infected mice, treatment with the hematopoietic growth factor, Fms-like tyrosine-kinase 3 ligand (Flt3 ligand) before infection reduced the lethality and tissue viral loads. Flt3 ligand failed to enhance the production of type I interferons. Instead, Flt3 ligand boosted the numbers of dendritic cells and, particularly lymphocytes in infected organs with an expansion of spleen B cells, and resulted in an increased titer of virus-specific antibody with neutralizing activity in the serum. The protective effect of Flt3 ligand was abolished in B cell-deficient mice. Our findings revealed that Flt3 ligand administration promotes resistance to EV-A71 infection with enhanced B cell response in a mechanism rarely reported before.

## Introduction

Enterovirus A71 (EV-A71), a member of the *Picornaviridae* family, infects humans by the fecal-oral route and induces mild symptoms, such as herpangina and hand-foot-and-mouth disease. The initial illness typically resolves, but sometimes is followed by fatal neurological manifestations, such as aseptic meningitis, brainstem encephalitis, encephalomyelitis, and acute flaccid paralysis with cardiopulmonary complications, which can cause severe long-term neurologic sequelae in survivors or death^1–3^. In the Asia-Pacific region, widespread and deadly EV-A71 outbreaks have been reported frequently for the last two decades, and EV-A71 is becoming an important pathogen for children^4,5^. China has developed vaccines, which are under testing and unavailable to other countries. Studies searching for anti-EV-A71 agents and their antiviral mechanisms are needed to control fatal infection.

Infants and young children are highly susceptible to fatal EV-A71 infection. As a rule, antiviral defenses of neonates are less effective than those of adults in fighting viral infections. Mouse studies found that interferon (IFN) production in neonatal hosts is inadequate to reduce viral replication^6–8^. In addition, the lymphocyte numbers of neonatal mice are 1,000–10,000 times less than those of adult mice^9,10^. With regard to EV-A71 infection, we reported that the responses of endogenous type I or type II IFNs, CD4 T cells, CD8 T cells, B cells, or antibody in mice provide resistance to infection by reducing tissue viral loads^11–13^.

Dendritic cells (DCs), an important component of both innate and adaptive immunity, exert their antiviral activities by priming T helper cells before activating B cells to produce antibodies, activating cytotoxic T cells, or producing IFNs^14–17^. Two main categories of DCs have been identified, and they appear to inhibit viral infections by different mechanisms. Plasmacytoid DCs (pDCs) mainly secrete type I IFNs (IFN-α) to clear virus in mouse tissues^16–18^. Conventional DCs (cDCs) primarily activate adaptive immunity to reduce viral loads in mouse tissues^19,20^. During the activation of adaptive immunity, a certain threshold of DCs is required; however, the number of DCs in the spleen of neonatal mice is 50 times less than that of adult mice^21^.

Fms-like tyrosine-kinase 3 ligand (Flt3 ligand) is a growth factor for hematopoietic precursor cells and can increase the numbers of B cells and DCs^7,22–24^. Previous studies assessed Flt3 ligand pretreatment in mice with respiratory syncytial virus (RSV) or lethal herpes simplex virus 1 (HSV-1) infection^7,8,25,26^ and concluded that Flt3 ligand exerts protection mainly through innate immune responses, type I IFNs and pDCs. Given that Flt3 ligand is capable of boosting both type I IFN and B cell responses, which effectively decrease EV-A71 infection^11,13^, we tested the efficacy of Flt3 ligand treatment on neonatal mice infected with EV-A71 in this study. Our results revealed that treatment with Flt3 ligand before infection significantly reduced the lethality and tissue viral loads of infected mice. Flt3 ligand pretreatment increased the number and antibody response of B cells, but failed to enhance the production of type I IFNs.

## Results

### Flt3 ligand pretreatment reduces the lethality and tissue viral loads of EV-A71-infected mice

**Figure 1:**
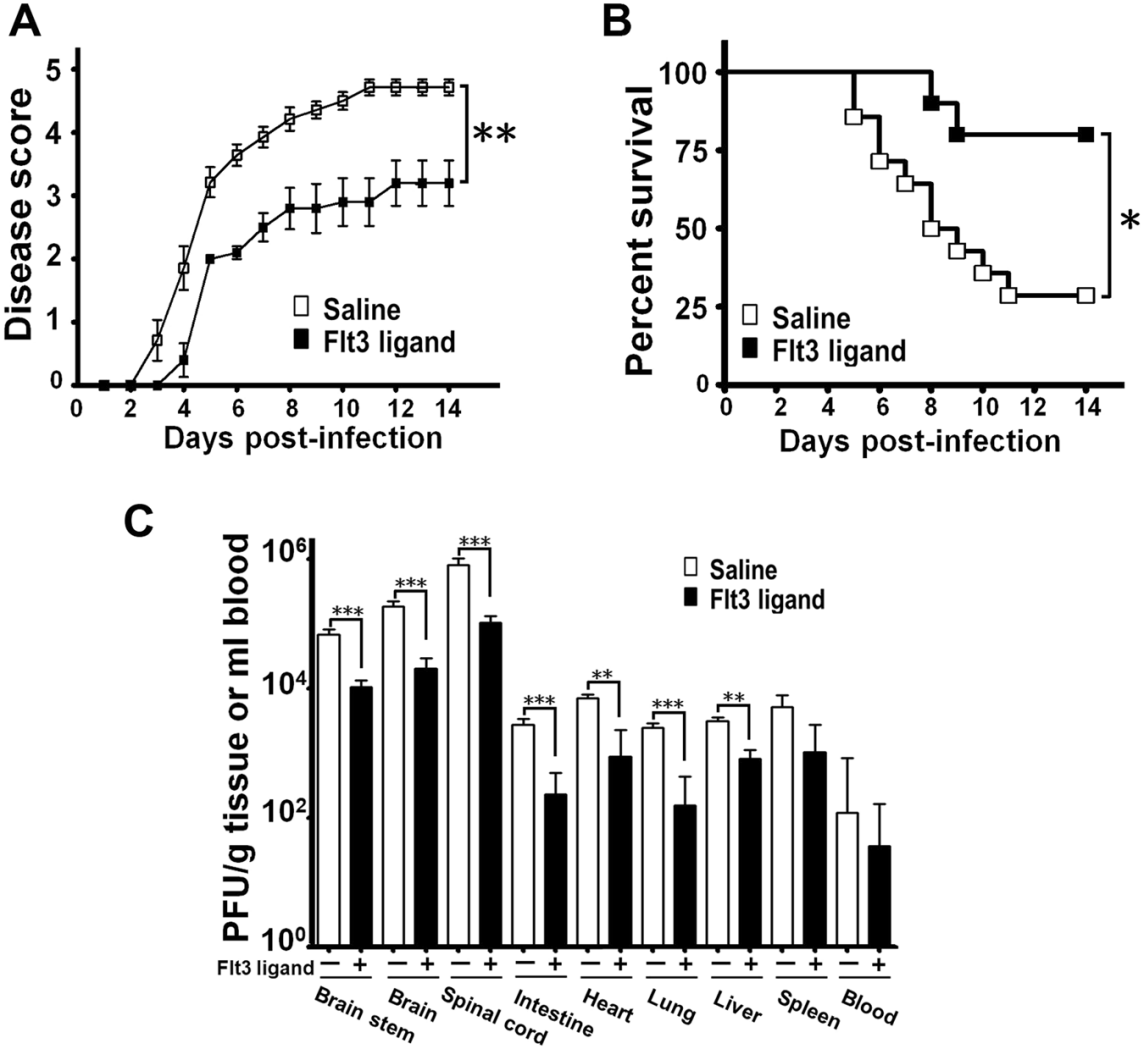
Flt3 ligand pretreatment reduces the morbidity, mortality, and tissue viral loads of EV-A71-infected mice. The disease scores (**A**) and survival rates (**B**) of infected mice, which were pretreated with saline (*n* = 14) or Flt3 ligand (*n* = 10), are shown. (**C**) The indicated tissues and organs of mice pretreated with saline (*n* = 6) or Flt3 ligand (*n* = 7) were harvested on day 7 post-infection to determine viral titers. In panels **A** and **C**, data represent means ± or + SEs. *P < 0.05; **P < 0.01; and ***P < 0.001.

We first assessed the efficacy of Flt3 ligand to protect neonatal mice from EV-A71 infection. One-day-old C57BL6/J mice were given one subcutaneous injection of recombinant mouse Flt3 ligand or saline daily for six days and then orally inoculated with virus one day after the last treatment. The infected mice pretreated with saline displayed signs of encephalitis, as manifested by hunch posture, lethargy, ataxia, and paralysis in both hind limbs, and succumbed to death before day 14 post-infection (p.i.) (Fig. 1A). Compared with the saline pretreatment, Flt3 ligand pretreatment significantly decreased the final death rate by 51% and the disease scores of infected mice (Fig. 1A, B) (*P* < 0.05). Flt3 ligand pretreatment also significantly reduced the mean viral titers in the central nervous system (brain stems, brains, and spinal cords) as well as peripheral organs (intestines, hearts, lungs, and livers) on day 7 p.i. (*P* < 0.01) by about 3- to 7-fold (Fig. 1C). Flt3 ligand pretreatment slightly reduced the mean viral titers in the spleen and blood.

We performed similar study on another strain of mice, ICR (Supplementary Fig. S1). Again, in infected mice, Flt3 ligand pretreatment significantly reduced the disease scores (*P* < 0.001), the final mortality rate (*P* < 0.05) by 44%, and the mean viral titers in several vital tissues or organs (*P* < 0.05) by about 10- to 330-fold when compared with the saline pretreatment group. Results obtained from two strains of mice suggest that the protective effect of Flt3 ligand against EV-A71 infection is not specific to a particular background of mice.

### Flt3 ligand pretreatment increases the numbers of DCs and lymphocytes in mouse organs

**Figure 2:**
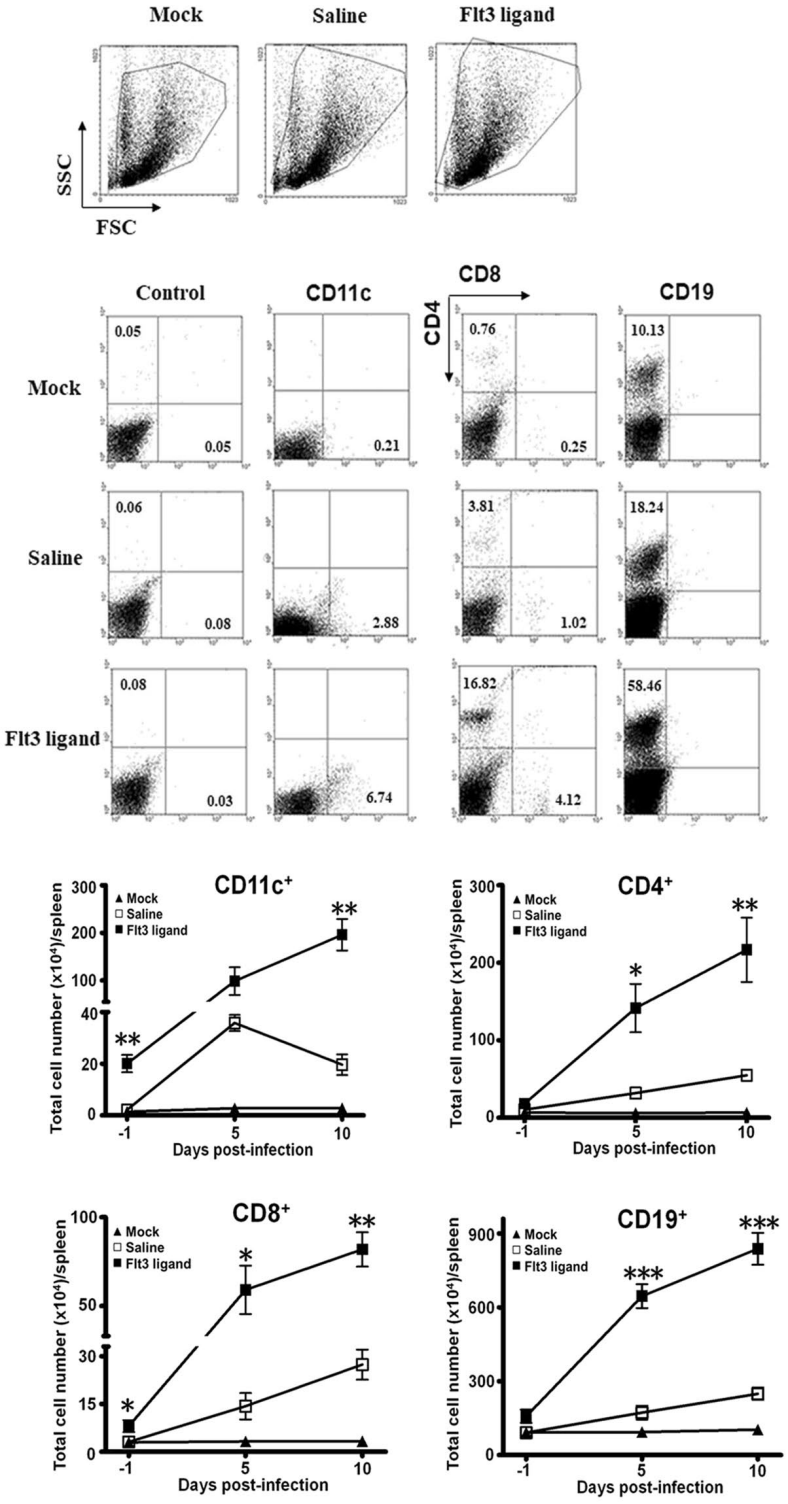
Flt3 ligand treatment increases the numbers of DCs and lymphocytes in the mouse spleen. Infected mice were pretreated with saline (Saline) or Flt3 ligand (Flt3 ligand). Mice without treatment and infection (Mock) served as a control. Splenocytes were analyzed by flow cytometry, the representative data are shown. The numbers of CD11c^+^ DCs, CD4^+^ T cells, CD8^+^ T cells, and CD19^+^ B cells in mouse spleens at the indicated times are shown. Data represent means ± SEs of 4 samples per data point. *P < 0.05; **P < 0.01; and ***P < 0.001 compared with the saline-treated groups at the same time point.

Flt3 ligand, a growth factor for hematopoietic precursor cells, is reported to increase DCs in the mouse spleen^7,26^ and lymphocytes in the mouse spleen and brain^22,23,27^. We detected EV-A71 in both mouse spleen and brain (Fig. 1C), therefore we quantified the numbers of DCs and lymphocytes in these two organs of C57BL6/J mice one day before infection (right after the last treatment of saline or Flt3 ligand) and also after infection using flow cytometry. The end time point for this study was set on day 10 p.i., when 36% infected mice in the saline-treated group were available for analyses. In the spleen and compared with the saline control, Flt3 ligand significantly increased the mean numbers of CD11c^+^ DCs (*P* < 0.01) before infection and on day 10 p.i. (Fig. 2). Flt3 ligand significantly increased the mean numbers of CD4^+^ T cells (*P* < 0.05) on days 5 and 10 p.i. (Fig. 2). Flt3 ligand significantly increased the mean numbers of CD8^+^ T cells (*P* < 0.05) before infection and on days 5 and 10 p.i. (Fig. 2). Flt3 ligand significantly increased the mean numbers of (CD19^+^) B cells (*P* < 0.001) on days 5 and 10 p.i. (Fig. 2). The number of B cells were more than those of CD4^+^ or CD8^+^ T cells in Flt3 ligand-treated mice on day 5 p.i. (*P* < 0.001).

In the mouse brain, leukocytes were rarely detected before EV-A71 infection regardless of whether mice were treated with Flt3 ligand or not (Supplementary Fig. S2). Viral infection increased the numbers of infiltrating (CD45^high^) leukocytes. Among CD45^high^ leukocytes, Flt3 ligand significantly increased the mean number of CD11c^+^ DCs on day 5 p.i. and the mean numbers of CD11c^+^ DCs, CD4^+^ T cells, and CD8^+^ T cells on day 10 p.i. in infected mice when compared with the saline control (*P* < 0.05). Flt3 ligand slightly increased B cell numbers after infection. Flow cytometric results collectively show that Flt3 ligand expands leukocytes in the spleen before and after infection and in the brain after infection.

We further characterized DCs by quantifying the numbers of cDCs and pDCs^28,29^ in the brain and spleen of infected mice on day 5 p.i., when abundant DCs were detected in both organs. Among CD45^high^ cells in the brain, Flt3 ligand increased the mean number of (CD11c^+^ MHC II^+^) cDCs (*P* = 0.05) and slightly boosted the mean number of (CD11c^+^ B220^+^) pDCs when compared with saline (Supplementary Fig. S3). Notably, the number of cDCs was about 3.5 times greater than that of pDCs in Flt3 ligand-treated mice. In the spleen, Flt3 ligand also slightly increased the numbers of both cDCs and pDCs (Supplementary Fig. S3).

### The effects of Flt3 ligand pretreatment on the survival of infected mice with deficiency in lymphocytes

**Figure 3:**
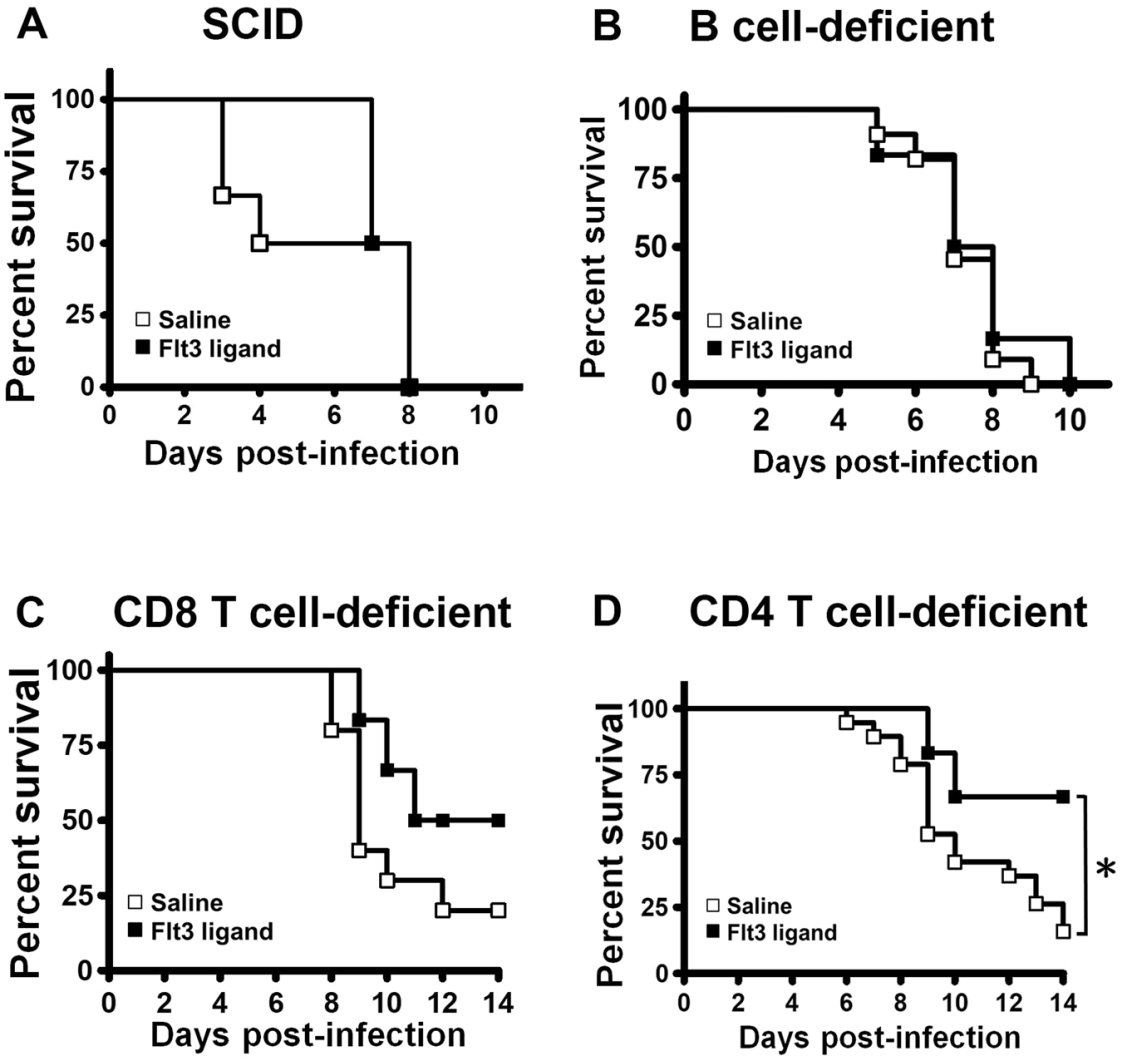
The effects of Flt3 ligand pretreatment on the survival of lymphocyte-deficient mice infected with EV-A71. The survival rates of infected SCID mice (**A**), B cell-deficient mice (**B**), CD8 T cell-deficient mice (**C**), and CD4 T cell-deficient mice (**D**) pretreated with saline (*n* = 6–19 per group) or Flt3 ligand (*n* = 6 per group) are shown. *P < 0.05.

Flow cytometric results showed that Flt3 ligand increases CD4, CD8, and B lymphocytes in infected mice. This finding led us to determine the importance of lymphocytes in Flt3 ligand-induced protection against EV-A71 lethality using lymphocyte-deficient mice. All severe combined immunodeficiency (SCID) mice deficient in both B and T cells died by day 8 p.i. regardless of whether the infected mice were pretreated with saline or Flt3 ligand (Fig. 3A), showing the significance of lymphocytes. We next analyzed the specific lymphocyte subset, which is important for Flt3 ligand to exert protective effect. All infected mice deficient in mature B cells died by day 10 p.i. regardless of whether the mice were pretreated with saline or Flt3 ligand (Fig. 3B). For mice deficient in CD8^+^ T cells, the survival rate of Flt3 ligand-treated mice was higher than that of saline-treated mice by 30% after infection (Fig. 3C). The difference in the survival rates between Flt3 ligand- and saline-treated groups was not statistically significant and less than those (44–55%) found in immunocompetent (wild-type) mice. For mice deficient in CD4^+^ T cells, the survival rate of Flt3 ligand-treated mice was significantly higher than that of saline-treated mice (*P* < 0.05) by 51% after infection (Fig. 3D), at a rate comparable to those observed in immunocompetent mice. Collectively, deficiency of B cells, but no CD4^+^ or CD8^+^ T cells, diminishes the protective effect of Flt3 ligand.

### Flt3 ligand pretreatment increases the production of virus-specific antibodies with neutralizing activity in mice

**Figure 4:**
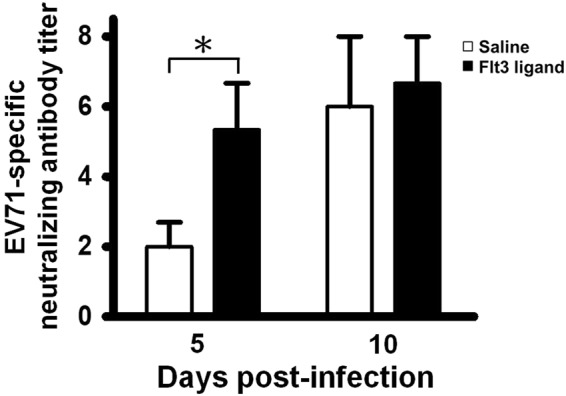
Flt3 ligand pretreatment increases the production of virus-specific antibodies with neutralizing activity in infected mice. Titers of neutralizing antibody titers in sera of infected mice pretreated with saline or Flt3 ligand are shown. Data represent means ± SEs of 3–10 samples per group. *P ≤ 0.05.

Here we reveal the essential role of B cells in protecting Flt3 ligand-treated mice from EV-A71-induced lethality. B cells primarily produce antibodies to fight viral infections. Our previous report showed that the endogenous antibody response protects mice from EV-A71 infection by reducing tissue viral loads^13^. We therefore studied the effect of Flt3 ligand pretreatment on antibody production using wild-type C57BL6/J mice (Fig. 4). The mean titer of neutralizing antibodies detected in sera of Flt3 ligand-treated mice was higher than that of saline-treated mice on day 5 p.i. (*P* < 0.05).

### Flt3 ligand pretreatment fails to increase the expression of type I IFNs in infected wild-type mice and to protect mice with type I IFN receptor deficiency from infection

**Figure 5:**
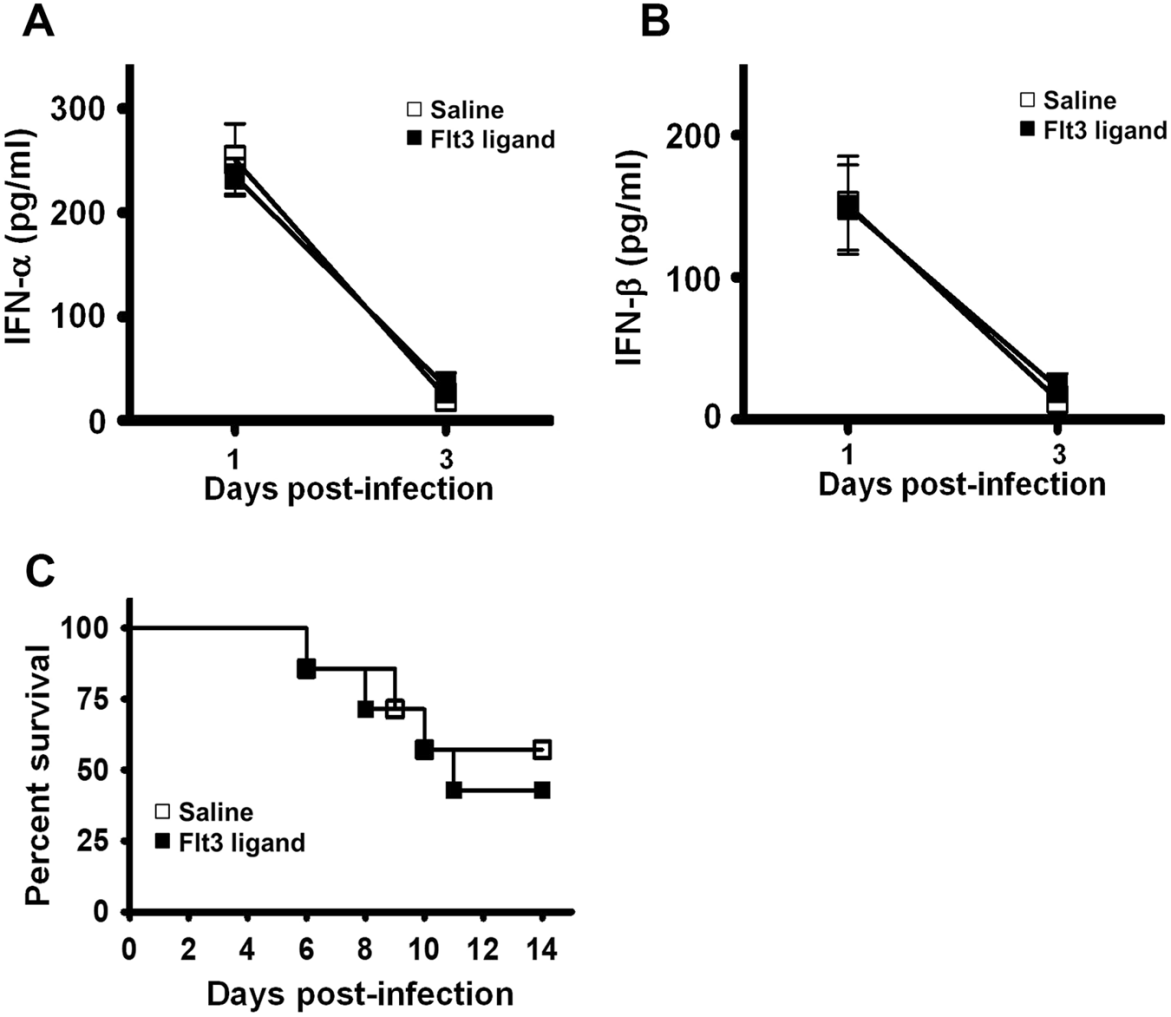
Flt3 ligand pretreatment fails to increase the expression of type I IFNs in infected wild-type mice and to protect mice with type I IFN receptor deficiency from infection. Infected C57BL6/J mice were pretreated with saline or Flt3 ligand. Levels of IFN-α (**A**) and IFN-β (**B**) in mouse sera at the indicated times are shown. Data represent means ± SEs of 3–4 samples per data point. (**C**) The survival rates of type I IFN receptor-deficient mice, which were pretreated with saline (*n* = 7) or Flt3 ligand (*n* = 7) before infection, are shown.

Flt3 ligand pretreatment is shown to protect mice from HSV-1-induced lethality through type I IFN, but not through lymphocytes, because deficiency of type I IFN receptor, but not lymphocytes, abolishes the protective effect^7^. Another previous report also highlighted the importance of type I IFNs, as Flt3 ligand treatment prior to neonatal RSV infection augments type I IFN signaling pathways^8^. However, these reports failed to address the effect of Flt3 ligand pretreatment on the expression of type I IFNs. We therefore investigated the influence of Flt3 ligand pretreatment on the expression of type I IFNs, as our previous report revealed the significance of endogenous type I IFNs in protecting mice against EV-A71-induced lethality by reducing mouse tissue viral loads and mortality rate^11^. Both IFN-α and IFN-β levels in the serum of infected mice pretreated with saline or Flt3 ligand were below detection at 6 and 12 hours p.i. The serum IFN-α levels of infected mice pretreated with saline or Flt3 ligand were comparable on days 1 and 3 p.i. (Fig. 5A). IFN-α levels in the brain and brain stem of infected mice pretreated with saline or Flt3 ligand were also comparable on day 5 p.i. (Supplementary Fig. S4). The serum IFN-β levels of infected mice pretreated with saline or Flt3 ligand were comparable on days 1 and 3 p.i. (Fig. 5B). These results showed the failure of Flt3 ligand pretreatment to enhance type I IFN expression. We tested mice with deficiency of type I IFN receptor and found that Flt3 ligand pretreatment failed to protect the mice from EV-A71 infection (Fig. 5C).

### Treatment of mice with Flt3 ligand after EV-A71 infection

**Figure 6:**
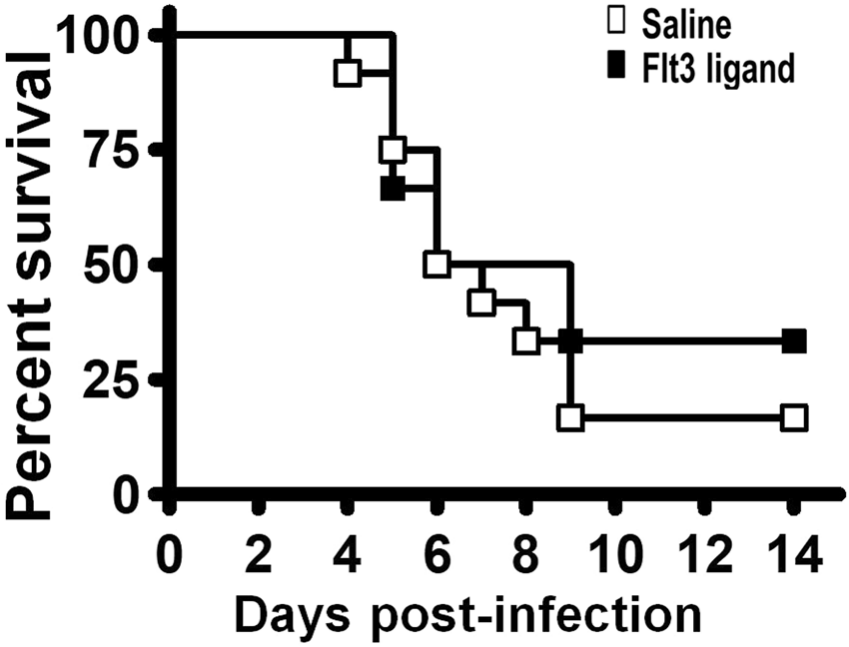
Treatment of mice with Flt3 ligand after infection. The survival rates of infected mice, which were treated with saline (*n* = 12) or Flt3 ligand (*n* = 6) on days 1–6 post-infection, are shown.

Lastly, we treated mice with Flt3 ligand after infection, as few reports have performed this test. Seven-day-old mice were infected with EV-A71 and given one subcutaneous injection of Flt3 ligand or saline daily from days 1 through 6 p.i. The survival rate of Flt3 ligand-treated mice was slightly higher than that of saline-treated mice by 20% (Fig. 6).

## Discussion

In the present study, we show that Flt3 ligand pretreatment reduces tissue viral loads and the lethality of EV-A71-infected mice. Flt3 ligand pretreatment increased the numbers of lymphocytes and DCs in infected tissues, but failed to affect the production of type I IFNs. B cells are a critical effector of Flt3 ligand-induced protection against EV-A71 infection, as Flt3 ligand boosts the spleen B cell number and the titer of virus-specific antibody with neutralizing activity and fails to protect B cell-deficient mice from infection.

Most of previous reports showing the antiviral mechanism of Flt3 ligand pretreatment in mice focused on the innate immunity^7,8,25^. The study of Vollstedt *et al*. concluded that Flt3 ligand exerts its antiviral effect through type I IFN- and NK-dependent innate immunity, because Flt3 ligand protected wild-type mice and lymphocyte-deficient mice, but not mice without type I IFN receptor or without both NK cells and lymphocytes, from HSV-1-induced death^7^. The study of Smit *et al*. found that Flt3 ligand decreased RSV-induced pathology with expansion of both pDCs and cDCs in the lung of adult mice^25^. The protection was mediated by pDC, as depletion of pDCs abolished the protective effect. The study of Remot *et al*. revealed that Flt3 ligand increased the DC number and type I IFN signaling pathways to limit the damage in the lung of mice upon primary infection or re-exposure to RSV^8^. In this report, the effect of Flt3 ligand on adaptive immunity was not fully addressed despite that Flt3 ligand protected mice from re-challenge with virus. Studies of Vollstedt *et al*. and Smit *et al*. failed to investigate the effect of Flt3 ligand on tissue viral burden, and the study of Remot *et al*. showed the failure of Flt3 ligand to affect tissue viral replication. All three previous reports failed to address the effect of Flt3 ligand on the production of type I IFNs despite their suggestion that type I IFNs or pDCs (a major producer of IFN-α) mediated the protective effect of Flt3 ligand. Our present study shows that Flt3 ligand fails to boost type I IFN expression, but deficiency of type I IFN receptor abrogates Flt3 ligand-induced protection, in infected mice. Despite a previous *in vitro* study showed that mouse DCs obtained by culture of bone marrow cells with Flt3 ligand could induce IFN-α secretion after EV-A71 infection^30^, our mouse *in vivo* results showed the failure of Flt3 ligand to increase serum IFN-α levels in infected mice. One possible explanation is that in addition to DCs, several other types of leukocytes, such as macrophages and nature killer cells, can produce IFN-α in infected mice.

Instead, Flt3 ligand boosts the spleen B cell number and titer of virus-specific antibodies with neutralizing activity in the serum of infected mice. These findings have not been reported before. Using lymphocyte-deficient mice, our result showing that Flt3 ligand fails to protect SCID mice deficient in both B and T cells from EV-A71 infection is different from that found in the study of Vollstedt *et al*., in which Flt3 ligand effectively prevents the lethality of RAG-deleted mice lacking mature B and T cells after HSV-1 infection^7^. It is general believed that B cells need help from CD4 T cells to induce antibody response. However, we found that deficiency of B cells, but not CD4 T cells, abolished the protective effect of Flt3 ligand, suggesting that innate B cells, but not T helper cells, might contribute to Flt3 ligand-mediated protection in our model. These results are consistent with our previous report demonstrating that CD4 T cell-deficient mice can produce neutralizing antibodies to protect mice from EV-A71 infection possibly through innate B cells^31^. Our additional results showing the failure of Flt3 ligand to increase the production of T helper 2 cytokines (IL-4 and IL-5) in EV-A71-infected mice (Supplementary Fig. S5), support our notion. In the future, the issue regarding how Flt3 ligand enhances responses of innate B cells in infected mice needs investigation. This also explains our result showing that Flt3 ligand pretreatment increases the production of antibodies at early time point (day 5 p.i.), but not at late time point (day 10, p.i.).

We also revealed that the absence of CD8 cells partially abrogated the protective effect of Flt3 ligand in EV-A71-infected mice. CD8 cells are a major producer of IFN-γ, which functions to protect mice from EV-A71 infection^13^. Our additional study revealed that Flt3 ligand pretreatment slightly enhanced IFN-γ levels in the central nervous system of infected mice (Supplementary Fig. S6). The present study leads to a better understanding of how Flt3 ligand suppresses viral infections with enhanced B cell responses. Overall, our study complements previous Flt3 ligand reports, which focused on innate immunity^7,8,25^, to illustrate that the protective mechanisms of Flt3 ligand against viral infections are manifold.

Regarding the pathogenesis of EV-A71 infection, the scientific and clinical communities have debated whether immature or excessive host immunity causes death or severe symptoms. Young children, who have inadequate, immature, and suppressed immune responses, are very susceptible to infection and likely to develop fatal symptoms after infection. Agents boosting lymphocyte numbers might help to fight infection, as infected patients with lymphopenia are at risk of developing severe complications^32,33^. However, clinical reports have found high levels of cytokine, chemokine, T cell, and antibody responses, particularly in patients with fatal symptoms and recommended anti-inflammatory therapies for treatment^34–37^. This uncertainty hinders the development of therapeutic agents to control fatal EV-A71 outbreaks. Our previous reports demonstrated that deficiency of endogenous type I or type II IFNs, CD4 T cells, CD8 T cells, B cells, or antibody response aggravates EV-A71 infection in mice with elevated tissue viral loads^11–13^. These findings argue against the notion that host immunity exacerbates infection. Our present study using Flt3 ligand, which protects mice from infection by boosting host B cell responses, and our previous report using an anti-inflammatory agent (dexamethasone), which increases the mortality of infected mice by reducing host immune responses^38^, further support the idea that the inadequate immunity renders the host susceptible to infection.

In the present study, we treated mice with ≤1 mg/kg/day of Flt3 ligand, which has been tested in clinical trials for cancer therapy and is well tolerated with little if any side effects even at doses up to 100 mg/kg/day for 14 days^39^. Because pretreatment yields protection against infection, it will be of interest to explore the potential of using Flt3 ligand for prophylactic treatment during outbreak in the future. Additionally, Flt3 ligand is shown to have adjuvant effect by enhancing cellular immunity against simian immunodeficiency virus or RSV infection^25,40^. Current murine models using wild-type mice for EV-A71 vaccine study are limited, because mice older than 14 days are relatively resistant to infection. When suitable animal models become available, future studies are needed to determine whether Flt3 ligand can be a vaccine adjuvant for boosting antibody response to prevent fatal EV-A71 infection.

## Methods

### Cell, virus, and mice

The human muscular (rhabdomyosarcoma, RD) cell line was maintained and propagated according to the instructions of American Type Culture Collection. EV-A71 strain M2 was propagated in and titrated on RD cell monolayers as previously described^41^. All animal methods and care described in the present study were performed in accordance with national guidelines and regulations. All mouse experiment protocols were approved by the Institutional Animal Care and Use Committee of National Cheng Kung University with the approval number of 105283. C57BL6/J mice, C57BL6/J-derived mice deficient in B cells (B6.129S2-*Igh-6*^*tm1Cgn*^/J), CD4 T cells (B6.129S2-*Cd4*^*tm1Mak*^/J), CD8 T cells (B6.129S2-*Cd8a*^*tm1Mak*^/J), or IFN (α and β) receptor 1 (B6.129S2-*Ifnar1*^*tm1Agt*^/Mmjax) (The Jackson Laboratory), and NOD.CB17-*Prkdc*^*scid*^ (SCID) mice were bred and maintained under specific pathogen-free conditions in the Laboratory Animal Center of National Cheng Kung University.

### Flt3 ligand treatment and infection of mice

Within 24 hours of birth, newborn mice were given one subcutaneous injection of phosphate-buffered saline or 1 μg/mouse (1 mg/kg) of recombinant mouse Flt3 ligand (R&D Systems) daily for 6 days in the scalp. One day after the last treatment, mice were infected with virus by oral inoculation as previously described^42^. Type I IFN receptor knockout mice were infected with 2 × 10^5^ plaque forming units/mouse, and the rest of mice were infected with 5 × 10^6^−2 × 10^7^ plaque forming units/mouse. We also treated infected mice with saline or Flt3 ligand from days 1 to 6 post-infection. Infected mice were monitored for signs of disease and survival. The disease score was graded as follow: 0, healthy; 1, ruffled hair; 2, weakness in hind limbs; 3, paralysis in single hind limb; 4, paralysis in both hind limbs; and 5, death. In separate experiments, mouse tissues were collected, processed, and subjected to plaque assay for titration of virus or to flow cytometry for quantifying leukocyte numbers.

### Leukocyte purification and flow cytometric assay

Leukocytes were isolated from mouse spleens, stained, and analyzed as previously described^43^ using FITC-, PE-, or Cy5-conjugated control antibodies or antibodies against mouse antigens, CD4 (clone GK 1.5), CD8a (clone 53–6.7), CD11c (clone N418), and CD19 (clone 6D5) purchased from eBioscience.

### Neutralization assay

The titers of EV-A71-specific antibodies with neutralizing activity in mouse sera were determined by neutralization assay as previously described using RD cell monolayers^13^. The highest dilution of serum that protected RD cell monolayers from infection was taken as the neutralizing titer.

### Measurement of IFNs by enzyme-linked immunosorbent assay (ELISA)

Mouse blood and brains were processed as previously described^12^ and subjected to ELISA to measure IFN-α or IFN-β using commercially available kits (R&D Systems).

### Statistical analyses

Data are expressed as mean ± SE values. For statistical comparison, disease score curves were analyzed by the Wilcoxon test, Kaplan-Meier survival curves were analyzed by the log-rank test, tissue viral titers were analyzed by the Mann-Whitney *U* test, and the rest of data were analyzed by the Student’s *t* test. All *P* values are for 2-tailed significance tests. A *P* value of ≤0.05 was considered statistically significant.

## Electronic supplementary material

Supplementary Information
