# Supplementary material for: Flt3 ligand treatment reduces enterovirus A71 lethality in mice with enhanced B cell responses
Source: Sci Rep. 2018 Aug 15;8:12184. doi: 10.1038/s41598-018-30631-2 (PMC6093920; doi:10.1038/s41598-018-30631-2)
Supplement: Supplementary file 1 — Supplementary Information [file 41598_2018_30631_MOESM1_ESM.pdf]

Flt3 ligand treatment reduces enterovirus A71 lethality in mice with enhanced B cell responses

Yu-Wen Lin[1], Li-Chiu Wang[1], Chien-Kuo Lee[2], Shun-Hua Chen[1,3]

[1]Institute of Biomedical Sciences and [3]Department of Microbiology and Immunology, College of Medicine, National Cheng Kung University, Tainan, Taiwan. [2]Institute of Immunology, College of Medicine, National Taiwan University, Taipei, Taiwan

Supplemental figures

Figure S1. Flt3 ligand pretreatment reduces the morbidity, mortality, and tissue viral loads of EV-A71-infected ICR mice.

Figure S2. Flt3 ligand pretreatment increases the numbers of DCs and lymphocytes in the brain of infected mice.

Figure S3. The effect of Flt3 ligand pretreatment on the numbers of cDCs and pDCs in tissues of infected mice.

Figure S4. Flt3 ligand pretreatment fails to increase the brain IFN-α level of infected mice.

Figure S5. Flt3L pretreatment fails to increase T helper 2 cytokine production in infected mice.

Figure S6. The effect of Flt3 ligand pretreatment on IFN-γ levels in the central nervous system of infected mice.

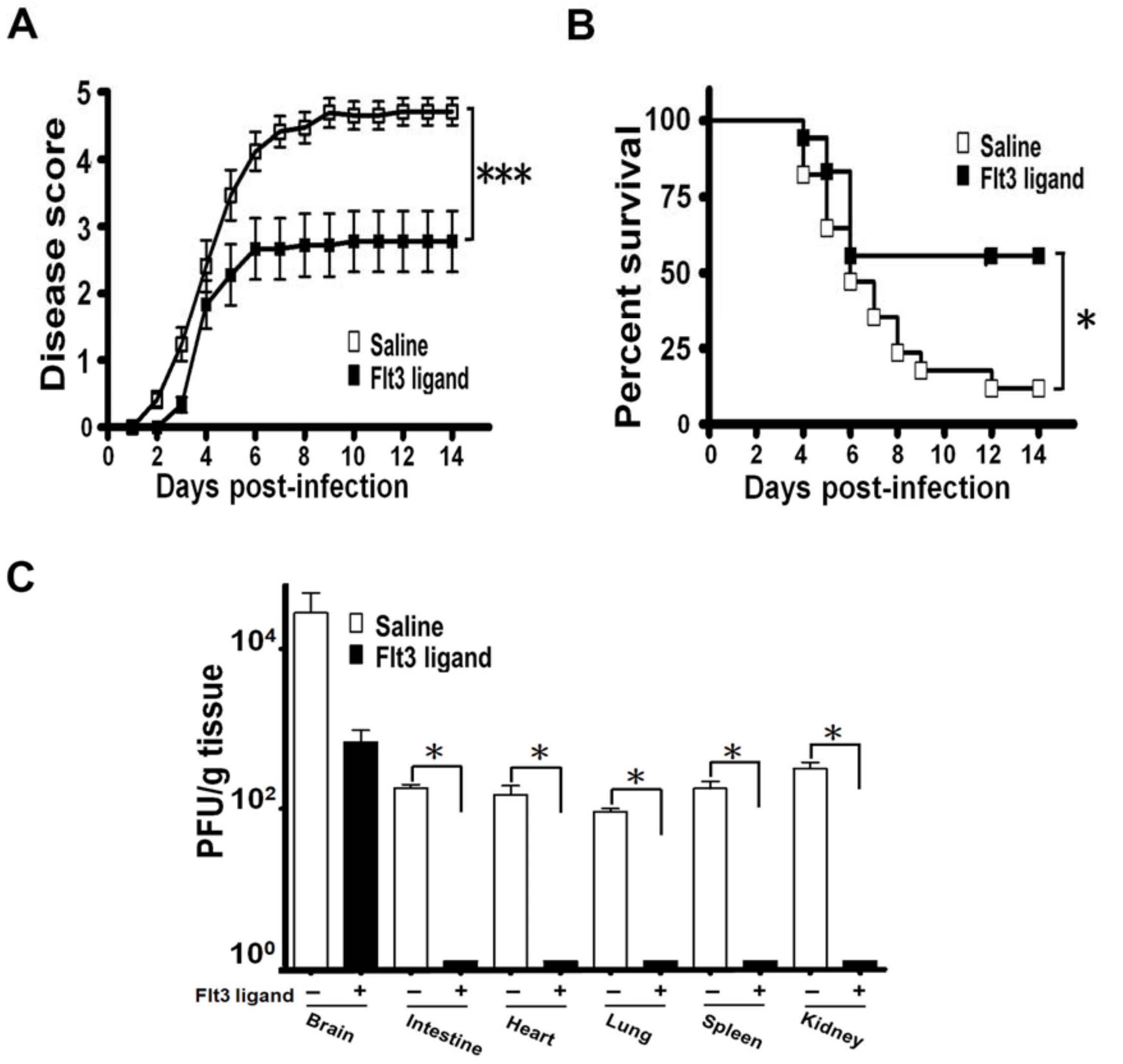

Figure S1. Flt3 ligand pretreatment reduces the morbidity, mortality, and tissue viral loads of EV-A71-infected ICR mice. The disease scores (**A**) and survival rates (**B**) of infected mice, which were pretreated with saline (*n* = 17) or Flt3 ligand (*n* = 18), are shown. (**C**) The indicated tissues and organs of mice pretreated with saline (*n* = 6) or Flt3 ligand (*n* = 7) were harvested on day 7 post-infection to determine viral titers. In panels **A** and **C**, data represent means ± or + SEs. *, $P < 0.05$; ** and ***, $P < 0.001$.

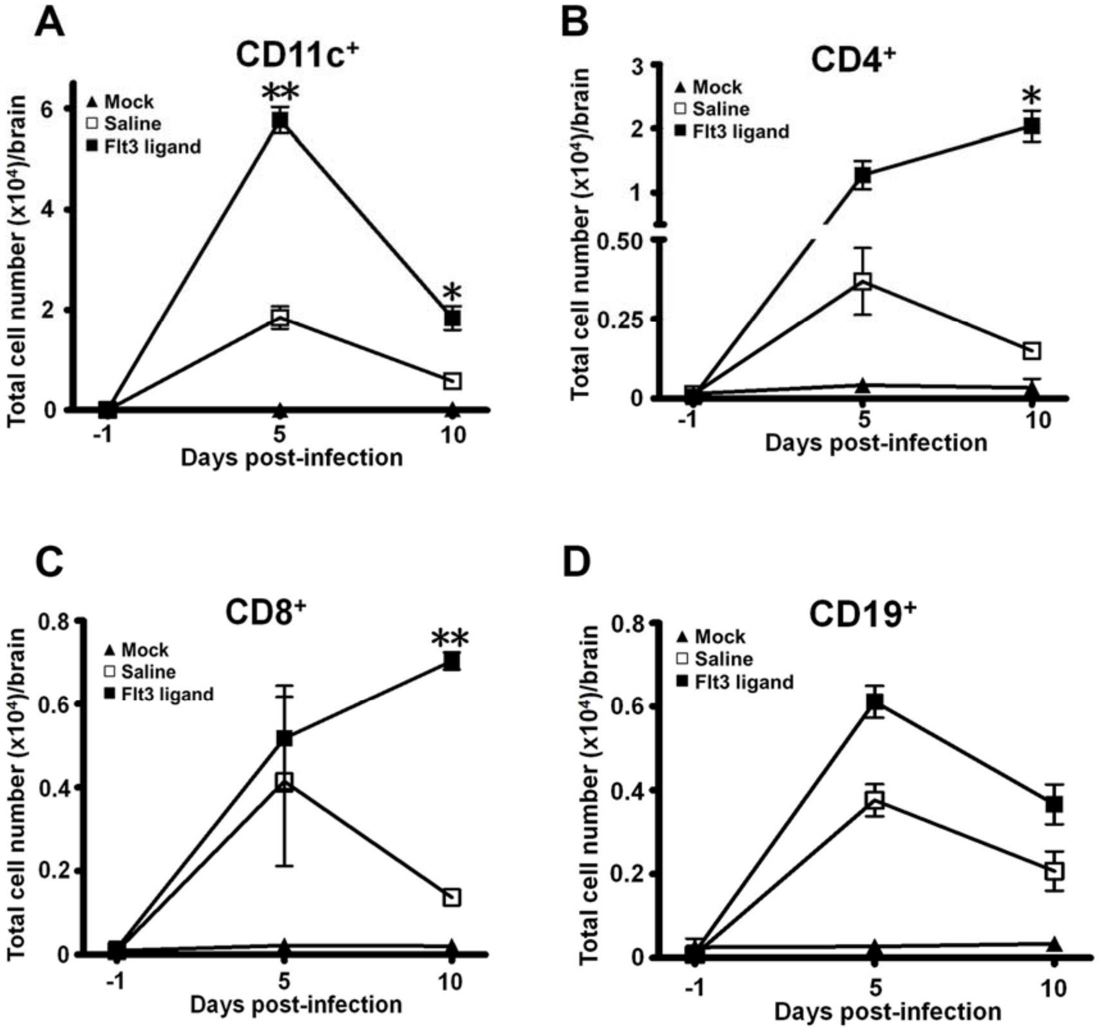

Figure S2. Flt3 ligand pretreatment increases the numbers of DCs and lymphocytes in the brain of infected mice. Infected mice were pretreated with saline (Saline) or Flt3 ligand (Flt3 ligand). Mice without treatment and infection (Mock) served as a control. Mouse brains were harvested, and leukocytes were prepared as previously described[13]. The numbers of $CD45^{high}$ $CD11c^{+}$ DCs (**A**), $CD45^{high}$ $CD4^{+}$ T cells (**B**), $CD45^{high}$ $CD8^{+}$ T cells (**C**), and $CD45^{high}$ $CD19^{+}$ B cells (**D**) in mouse brains at the indicated times are shown. The antibody against mouse antigen CD45 (clone 30-F11) purchased from eBioscience was used. Data represent means ± SEs of >3 samples per data point. *, $P < 0.05$ and **, $P < 0.01$ compared with the saline-treated groups at the same time point.

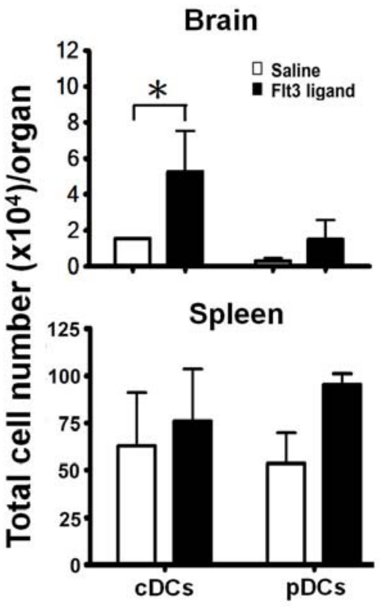

Figure S3. The effect of Flt3 ligand pretreatment on the numbers of cDCs and pDCs in tissues of infected mice. Mice were treated with saline or Flt3 ligand before infection. The numbers of ($CD45^{high}$ MHC II^+ $CD11c^+$) cDCs and ($CD45^{high}$ $B220^+$ $CD11c^+$) pDCs in mouse brains (top panel) and (MHC II^+ $CD11b^+$ $CD11c^+$) cDCs and ($B220^+$ $CD11c^+$) pDCs in mouse spleens (bottom panel) were determined on day 5 post-infection. Antibodies against mouse antigens, B220 (clone RA3-6B2) and MHC II (clone M5/114.15.2) purchased from eBioscience were used. Data represent means + SEs of >3 samples per group.

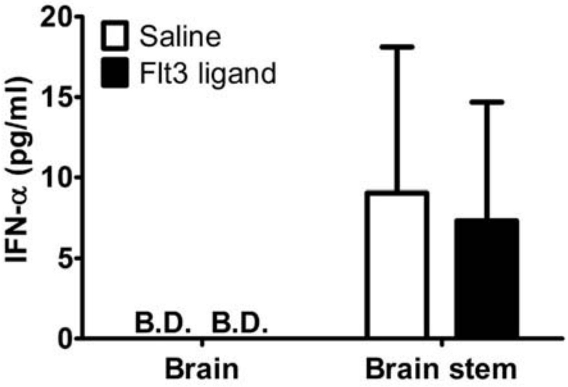

Figure S4. Flt3 ligand pretreatment fails to increase the brain IFN-α level of infected mice. Infected mice were pretreated with saline or Flt3 ligand. IFN-α levels in mouse brains and brain stems on day 5 post-infection are shown. Data represent means + SEs of >3 samples per group. B.D., below detection.

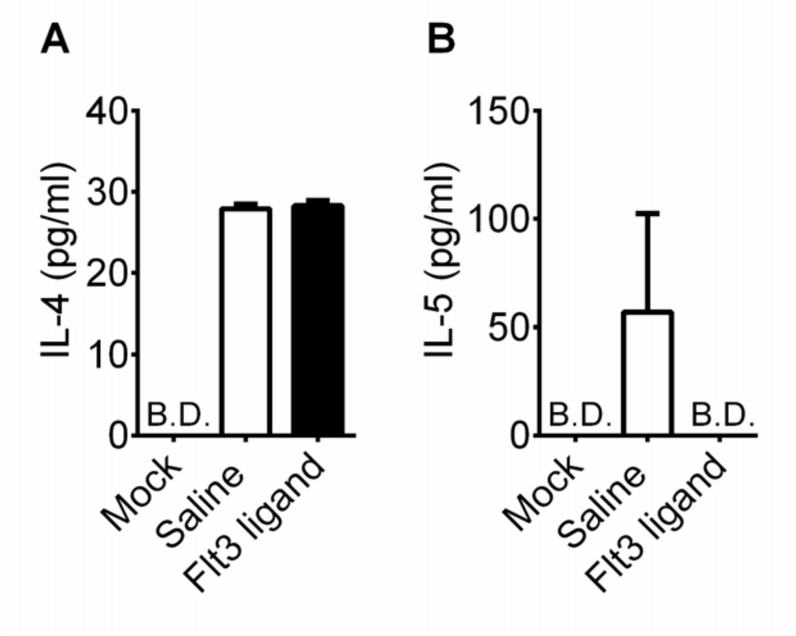

Figure S5. Flt3L pretreatment fails to increase T helper 2 cytokine production in infected mice. Infected mice were pretreated with saline (Saline) or Flt3 ligand (Flt3 ligand). Mice without treatment and infection (Mock) served as a control. Serum levels of IL-4 (A) and IL-5 (B) on day 7 post-infection were measured using commercially available ELISA kits (R&D Systems). Data represent means + SEs of 3-4 samples per group.

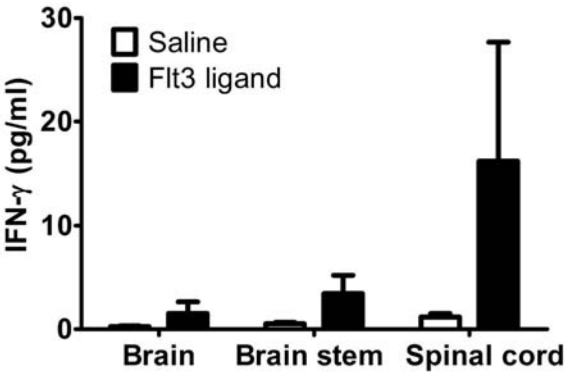

Figure S6. The effect of Flt3 ligand pretreatment on IFN-γ levels in the central nervous system of infected mice. Infected mice were treated with saline or Flt3 ligand. IFN-γ levels in the mouse central nervous system on day 7 post-infection were measured using a commercially available ELISA kit (R&D Systems). Data represent means + SEs of >3 samples per group.
